# Expression of multiple cancer-testis antigen genes in gastrointestinal and breast carcinomas

**DOI:** 10.1054/bjoc.2001.1974

**Published:** 2001-09

**Authors:** K Mashino, N Sadanaga, F Tanaka, H Yamaguchi, H Nagashima, H Inoue, K Sugimachi, M Mori

**Affiliations:** Department of Surgery, Medical Institute of Bioregulation, Kyushu University, 4546 Tsurumibaru, Beppu, 874-0838; Department of Surgery and Science, Graduate School of Medical Science, Kyushu University, 3-1-1 Maidashi, Higashi-ku, Fukuoka, 812-0054, Japan

**Keywords:** *MAGE*, tumour-rejection antigens, cancer-testis antigen, immunotherapy, cancer vaccine

## Abstract

Cancer-testis antigens (CTAs) such as *MAGE* are selectively expressed in various types of human neoplasms but not in normal tissues other than testis. This characteristic feature of CTAs makes them promising antigens for cancer-specific immunotherapy. A critical requirement for this therapy is identification of promising antigens. In this study, we investigated the expression of 6 genes recently identified by serological analysis of antigens by recombinant expression (SEREX) libraries: *NY-ESO-1*, *LAGE-1*, *SCP-1*, *SSX-1*, *SSX-2*, and *SSX-4*, in many surgical samples of gastrointestinal and breast carcinomas using reverse transcription-polymerase chain reaction. We found relatively high expression of *SCP-1* (23.5%) and *SSX-4* (20.6%) in gastric carcinoma, *LAGE-1* (39.1%) and *NY-ESO-1* (23.9%) in oesophageal carcinoma, and *SCP-1* (34.1%) in breast carcinoma. We also found frequent synchronous expression with *MAGE*, including *LAGE-1* (46.2%) in oesophageal carcinoma, *SSX-4* (46.7%) in gastric carcinoma, and *SCP-1* (38.3%) in breast carcinoma. Immunohistochemical analysis of the tumour samples expressing both *MAGE-4* and *NY-ESO-1* genes demonstrated differences in distribution between *MAGE-4* and *NY-ESO-1* in serial sections. We concluded that *NY-ESO-1*, *LAGE-1*, *SCP-1* and *SSX-4* genes may be promising candidates for cancer-specific immunotherapy in addition to *MAGE*, and that polyvalent cancer vaccines may be useful in cases of heterogeneous expressions of CTA genes in gastrointestinal and breast carcinomas. © 2001 Cancer Research Campaign http://www.bjcancer.com


BJOC 01-1974 713-720 20/8/01 4:20 pm Page 713

*British Journal of Cancer* (2001) **85**(5), 713–720
© 2001 Cancer Research Campaign
doi: 10.1054/ bjoc.2001.1974, available online at http://www.idealibrary.com on IDEAL

http://www.bjcancer.com

### Expression of multiple cancer-testis antigen genes in gastrointestinal and breast carcinomas

**K Mashino[1], N Sadanaga[1], F Tanaka[1], H Yamaguchi[1], H Nagashima[1], H Inoue[1], K Sugimachi[2] and M Mori[1]**

[1]Department of Surgery, Medical Institute of Bioregulation, Kyushu University, 4546 Tsurumibaru, Beppu, 874-0838; [2]Department of Surgery and Science, Graduate School of Medical Science, Kyushu University, 3-1-1 Maidashi, Higashi-ku, Fukuoka, 812-0054, Japan

**Summary** Cancer-testis antigens (CTAs) such as *MAGE* are selectively expressed in various types of human neoplasms but not in normal tissues other than testis. This characteristic feature of CTAs makes them promising antigens for cancer-specific immunotherapy. A critical requirement for this therapy is identification of promising antigens. In this study, we investigated the expression of 6 genes recently identified by serological analysis of antigens by recombinant expression (SEREX) libraries: *NY-ESO-1*, *LAGE-1*, *SCP-1*, *SSX-1*, *SSX-2*, and *SSX-4*, in many surgical samples of gastrointestinal and breast carcinomas using reverse transcription-polymerase chain reaction. We found relatively high expression of *SCP-1* (23.5%) and *SSX-4* (20.6%) in gastric carcinoma, *LAGE-1* (39.1%) and *NY-ESO-1* (23.9%) in oesophageal carcinoma, and *SCP-1* (34.1%) in breast carcinoma. We also found frequent synchronous expression with *MAGE*, including *LAGE-1* (46.2%) in oesophageal carcinoma, *SSX-4* (46.7%) in gastric carcinoma, and *SCP-1* (38.3%) in breast carcinoma. Immunohistochemical analysis of the tumour samples expressing both *MAGE-4* and *NY-ESO-1* genes demonstrated differences in distribution between *MAGE-4* and *NY-ESO-1* in serial sections. We concluded that *NY-ESO-1*, *LAGE-1*, *SCP-1* and *SSX-4* genes may be promising candidates for cancer-specific immunotherapy in addition to *MAGE*, and that polyvalent cancer vaccines may be useful in cases of heterogeneous expressions of CTA genes in gastrointestinal and breast carcinomas. © 2001 Cancer Research Campaign http://www.bjcancer.com

**Keywords**: *MAGE,* tumour-rejection antigens, cancer-testis antigen, immunotherapy, cancer vaccine

### INTRODUCTION

Gastrointestinal and breast carcinomas are the most common cancers and are responsible for the largest numbers of cancer-related deaths in the world (Greenlee et al, 2000; Ries et al, 2000). However, therapeutic options for the treatment of patients with these tumours are limited to 3 fundamental modalities: surgical resection, radiation therapy and chemotherapy. Against advanced carcinomas, therapeutic options are limited to radiation therapy and chemotherapy, and these modalities do not yield results. Cancer-specific immunotherapy may be expected to become a novel treatment modality for gastrointestinal and breast carcinomas.

Many genes coding tumour rejection antigens such as *MAGE* (Van der Bruggen et al, 1991), *BAGE* (Boel et al, 1995) and *GAGE* (Van den Eynde et al, 1995) have been isolated from melanoma cell lines. These antigens are recognized by autologous cytotoxic T-lymphocytes (CTLs), which are restricted by human leukocyte antigen (HLA) class I molecules, and some antigenic peptides have been identified (Van den Eynde et al, 1997). Furthermore, these antigens are also reported to induce class II restricted T-cell responses (Chaux et al, 1999; Manici et al, 1999). Some of these antigens are expressed in various tumours of different histological origins, but not in normal tissues other than testis. Therefore, these antigens have been designated cancer-testis antigens (CTAs) (Gure et al, 1997) and their characteristics make them promising candidates for cancer-specific immunotherapy; clinical trials using peptides such as MAGE-1 or MAGE-3 are in progress for malignant melanoma (Mukherji et al, 1995; Marchand et al, 1995).

We previously reported that *MAGE*-1 and *MAGE*-3 genes were expressed in 62% and 57% of oesophageal carcinomas (Inoue et al, 1997), 41% and 38% of gastric carcinomas (Inoue et al, 1995), 30% and 20% of colorectal carcinomas (Mori et al, 1996), 24% and 31% of breast carcinomas (Fujie et al, 1997), and more than 65% of hepatocellular carcinomas (Tahara et al, 1999). We also identified some MAGE gene-encoded peptides recognized by CTL (Tanaka et al, 1997; Fujie et al, 1999). Based on these findings, cancer-specific immunotherapy using the HLA class I restricted MAGE peptide has already begun in our institute for the treatment of gastrointestinal or breast carcinoma. Although this type of therapy appears promising and has thus far resulted in few side effects, application of this therapy is restricted by the tumour expression of the *MAGE* gene and patient HLA type.

Recently, a novel method has been established based on the specific recognition of autologous sera of some cancer patients, termed serological analysis of antigens by recombinant expression cloning (SEREX) (Sahin et al, 1995). This new method permits direct molecular determination of new tumour antigens that elicit an IgG antibody response in tumour patients. The method has enabled the discovery of several novel genes with tumour specificity, such as *NY-ESO-1* (Chen et al, 1997), *LAGE*. (Lethe et al, 1998), *SCP-1* (Türeci et al, 1998b), *SSX* (Türeci et al, 1996) and *CT7* (Chen et al, 1998). These antigens are also expected to become new candidates for cancer-specific immunotherapy, but little information is available on the comprehensive expression of CTAs in a large number of samples of gastrointestinal and breast carcinomas. In the present study, we compared the expression of newly found CTA genes with that of the *MAGE* gene in gastrointestinal and breast carcinomas, and investigated which CTAs are

*Received 19 January 2001*
*Revised 30 April 2001*
*Accepted 15 May 2001*

*Correspondence to:* M Mori

**Table 1** PCR amplification programs

| Gene | | Primers from 5′ to 3′ | Temperature and duration<br>Denaturation<br>Annealing<br>Extension | Cycle No. | Product. size |
|---|---|---|---|---|---|
| MAGE-1 | f<br>r | CGGCCGAAGGAACCTGACCCAG<br>GCTGGAACCCTCACTGGGTTGCC | 94˚C for 1 min<br>72˚C for 1 min<br>72˚C for 2 min | 33 | 421 bp |
| MAGE-3 | f<br>r | TGGAGGACCAGAGGCCCCC<br>GGACGATTATCAGGAGGCCTGC | 94˚C for 1 min<br>72˚C for 1 min<br>72˚C for 2 min | 33 | 725 bp |
| MAGE-4 | f<br>r | GAGCAGACAGGCCAACCG<br>AAGGACTCTGCGTCAGGC | 94˚C for 30 s<br>68˚C for 30 s<br>72 C for 30 s | 30 | 446 bp |
| NY-ESO-1 | f<br>r | CGCCTGCTTGAGTTCTACCTC<br>AGGGAAAGCTGCTGGAGACAG | 94˚C for 1 min<br>59˚C for 1 min<br>72˚C for 1 min | 30 | 218 bp |
| LAGE-1 | f<br>r | GCAGGATGGAAGGTGCCC<br>CTGGCCACTCGTGCTGGGA | 94˚C for 1 min<br>62˚C for 1 min<br>72˚C for 1 min | 28 | 399 bp |
| SCP-1 | f<br>r | GAAGAAAGGATGTTGACACAAAT<br>GTTTTTCCATAAGTGCTACCATT | 94˚C for 1 min<br>54˚C for 1 min<br>72˚C for 1 min | 42 | 487 bp |
| SSX-1 | f<br>r | CTAAAGCATCAGAGAAGAGAAGC<br>AGATCTCTTATTAATCTTCTCAGAAA | 94˚C for 1 min<br>54˚C for 1 min<br>72˚C for 1 min | 32 | 422 bp |
| SSX-2 | f<br>r | GTGCTCAAATACCAGAGAAGATC<br>TTTTGGGTCCAGATCTCTCGTG | 94˚C for 1 min<br>54˚C for 1 min<br>72˚C for 1 min | 32 | 435 bp |
| SSX-4 | f<br>r | AAATCGTCTATGTGTATATGAAGC<br>GGGTCGCTGATCTCTTCATAAAC | 94˚C for 1 min<br>56˚C for 1 min<br>72˚C for 1 min | 32 | 415 bp |
| GAPDH | f<br>r | GTCAACGGATTTGGTCGTATT<br>AGTCTTCTGGGTGGCAGTGAT | 94˚C for 1 min<br>56˚C for 1 min<br>72˚C for 1 min | 24 | 540 bp |

f: forward primer r: reverse primer.

useful candidates for cancer-specific immunotherapy for gastrointestinal and breast carcinomas.

### MATERIALS AND METHODS

### Cell lines

The fibrosarcoma cell line HT1080 was kindly provided by the Institute of Development, Aging and Cancer, Tohoku University, Sendai, Japan. The gastric carcinoma cell line Ns-8, and the choriocarcinoma cell lines SCH, were supplied by the Japanese Cancer Research Bank, Tokyo, Japan. The melanoma cell lines MZ-2, and LB-373-MEL, were kindly provided by Dr Bernard Lethe, Ludwig Institute for Cancer Research Brussels Branch, Bruxelles, Belgium.

### Tissue samples

Tumour samples (46 oesophageal squamous cell carcinomas, 102 gastric adenocarcinomas, 98 colorectal adenocarcinomas, and 129 breast adenocarcinomas) and matched control samples of the normal tissue located 5 cm away from the tumour edge were frozen immediately in liquid nitrogen less than 5 min after surgical resection and kept at –90˚C until RNA extraction. The surgical samples were obtained from the Department of Surgery, Medical Institute of Bioregulation, Kyushu University, Beppu, Department of Surgery, Oita Prefectural Hospital, Oita, and the Department of Surgery, Saitama Cancer Center, Saitama, Japan.

### Extraction of RNA and RT-PCR analysis

The acid guanidium thiocyanate-phenol-chloroform extraction procedure (Chomczynski and Sacchi, 1987) was used for extraction of total RNA. The cDNA was synthesized from 8.0 μg of total RNA as described previously (Inoue et al, 1995). The presence of *MAGE-1* and *MAGE-3* cDNA in the reverse transcription products was detected by polymerase chain reaction (PCR) amplification in separate reactions, using oligonucleotide primers located in the different exons of each gene (Weynants et al, 1994). The presence of *NY-ESO-1*, *LAGE-1*, *SSX-1*, *SSX-2*, *SSX-4* and *SCP-1* were determined by PCR amplification under the same conditions as reported previously (Lethe et al, 1998, Türeci et al, 1998a, Türeci et al, 1998b). Briefly a 1/100 aliquot of reverse transcription products were amplified in a 30 μl reaction mixture containing 10 nmoles of each dNTP (dATP, dTTP, dCTP, dGTP.), 1 μl of each oligonucleotide primer at 10 μM, 3 μl of 10 × PCR buffer (Perkin–Elmer, Branchburg, NJ, USA), and 1 U of AmpliTaq DNA polymerase (Perkin–Elmer). In the detection of *SCP-1*, 2 U

 *© 2001 Cancer Research Campaign*

BJOC 01-1974 713-720 20/8/01 4:20 pm Page 715

of AmpliTaq Gold (Perkin–Elmer) were substituted for AmpliTaq DNA polymerase. The reaction mixtures were then subjected to the appropriate PCR programmes as listed in Table 1. To confirm the specificity of the PCR products of the genes, we cloned the PCR product into pCRII vector (Invitrogen, San Diego, CA, USA) and then sequenced the cDNA by using the chain-termination DNA sequencing method. We then determined the nucleotide sequence of representative samples of PCR products and confirmed them to be identical to the expected fragments of cDNA in each CTA gene. An 8 μl aliquot of each PCR product was separated on a 1.5% agarose gel and visualized with ethidium bromide staining. The pattern of expression of the CTA genes was established with reverse transcription-PCR (RT-PCR) assays by evaluating the intensity of a band in agarose gels; if the band was recognized, the case was positive. The integrity of the RNA was confirmed by performing PCR amplification of each cDNA with

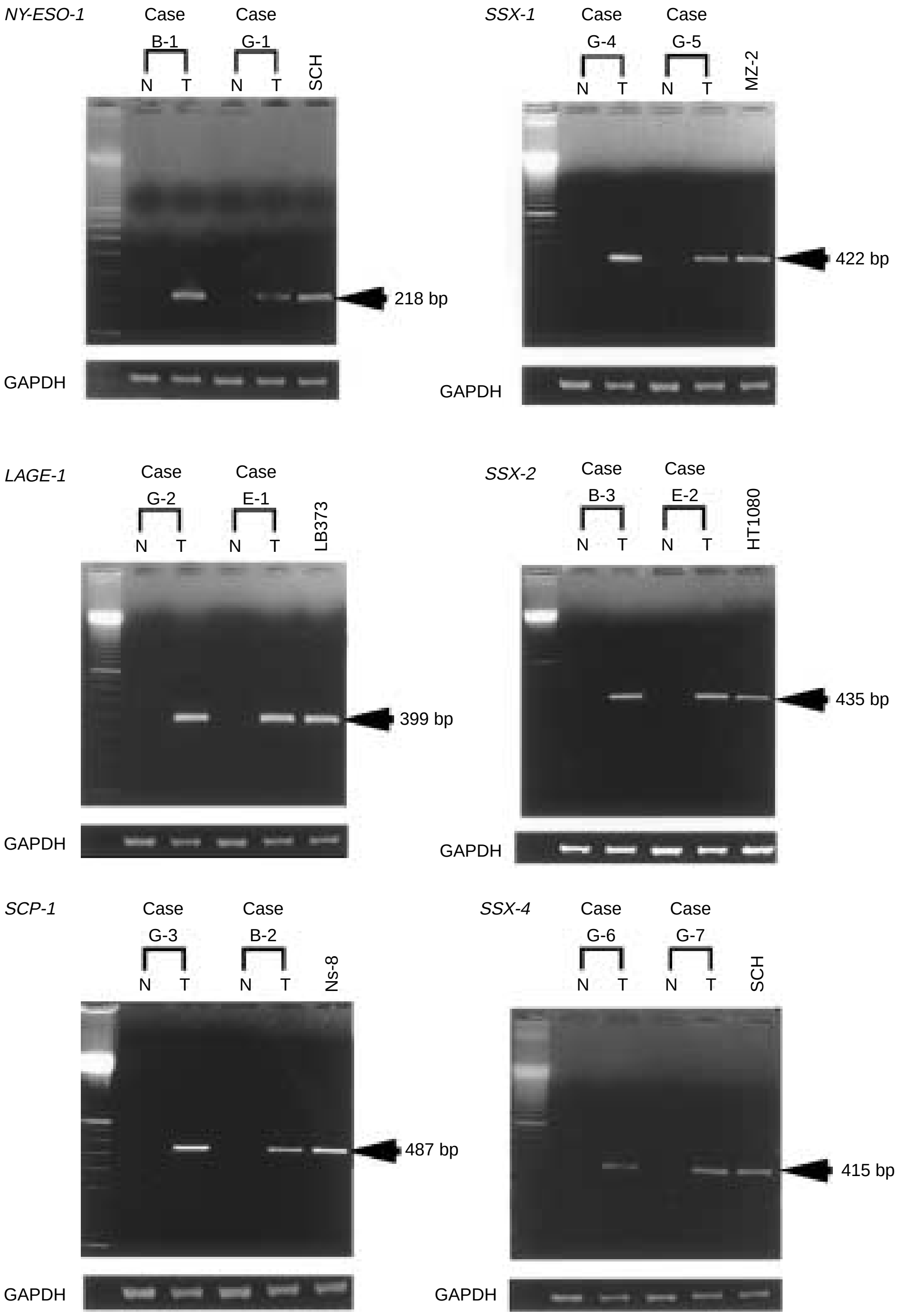

**Figure 1** Detection of *NY-ESO-1*, *LAGE-1*, *SCP-1*, *SSX-1*, *SSX-2* and *SSX-4* mRNA expression by RT-PCR in 2 representative cases of oesophageal carcinoma (Cases E-1, 2), 7 cases of gastric carcinoma (Cases G-1–7), and 3 cases of breast carcinoma (Cases B-1–3). cDNA extracted from tumour tissue (T) and paired normal tissue (N) was amplified in each case. Only tumour sites expressed CTA genes and there was no sample with expression of CTA genes in a normal site. GAPDH mRNA expression is shown as the corresponding control in the lower panel. Only positive cases were chosen to be presented

*© 2001 Cancer Research Campaign* 

primers for the gene glyceraldehyde-3-phosphate dehydrogenase (GAPDH) (Tokunaga et al, 1987).

### Immunohistochemistry

The primary antibodies used in this study were 57B and B9.8 mAb. Both were kindly provided by Dr GC Spagnoli (Kocher et al, 1995, Schultz et al, 2000). 57B mAb stains tumour tissues that express *MAGE-4* (Landry et al, 2000), and B9.8 mAb stains tumour tissues that express *NY-ESO-1* (Schultz et al, 2000). Frozen tumour sections were thawed, washed in PBS, fixed in formalin and incubated overnight at 4˚C in the presence of hybridoma supernatant from 57B or B9.8. For both preparations, bound antibodies were visualized using a Dako LSAB Peroxidase Kit (Dako), according to the manufacturer's recommendations, and cell nuclei were counterstained with hematoxylin.

### Clinico-pathologic data

All data including sex, histology, tumour size, depth of tumour invasion, lymph node metastasis, lymph vessel permeation, vascular vessel permeation, and disease stage were obtained from the clinical and pathologic records. Disease stage was classified according to the criteria proposed by the Japanese Society of Oesophageal Disease (8th edn), the Japanese Research Society of Gastric Cancer (12th edn), the Japanese Research Society of Colon Cancer (5th edn), and the Japanese Breast Cancer Society (12th edn). Tumours with or without expression of the CTA genes were then compared.

### Statistical analysis

Statistical analysis was performed by the chi-square or Fisher's exact test. The level of significance was set at $P < 0.05$.

### RESULTS

### Expression of CTA genes: cultured cell lines and clinical samples

The expression of 6 CTA genes, *NY-ESO-1*, *LAGE-1*, *SCP-1*, *SSX-1*, *SSX-2* and *SSX-4*, was determined by RT-PCR analysis. Each CTA was expressed in various types of carcinoma cell line. *NY-ESO-1* and *SSX-4* were expressed in SCH, *LAGE-1* in LB-373, *SCP-1* in Ns-8, *SSX-1* in MZ-2, and *SSX-2* in HT1080. These cell lines were used as positive controls in each analysis. In clinical samples, all 6 CTA genes were expressed exclusively in carcinoma tissues. None of the 6 CTA genes were expressed in the paired normal tissues (Figure 1).

### Frequency of expression of CTA genes in tumour samples

Expression of the 6 CTA genes was examined in 46 samples of oesophageal carcinoma, 102 samples of gastric carcinoma, 98 samples of colorectal carcinoma, and 129 samples of breast carcinoma. There was no CTA gene expressed more frequently than the *MAGE-1* and *MAGE-3* genes. We found relatively high expression of *LAGE-1* (39.1%) in oesophageal carcinoma, *SCP-1* (23.5%) and *SSX-4* (20.6%) in gastric carcinoma, and *SCP-1* (34.1%) in breast carcinoma, while we found high expression of none of the CTA genes including *MAGE-1* and *MAGE-3* in colorectal carcinoma. Table 2 summarizes the results of RT-PCR analysis of the CTA gene expression.

Expression of the 6 CTA genes correlated with none of the clinico-pathological parameters examined. There was no significant relationship between the expression of the 6 CTAs and clinical parameters for any type of carcinoma (data not shown).

### Synchronous expression of multiple CTA genes in the same tumour sample

Next, we analyzed multiple expression of the 8 CTA genes including *MAGE-1* and *MAGE-3* in each case. As shown in Figure 2A, synchronous expression of *MAGE-1*, *MAGE-3*, *NY-ESO-1*, and *LAGE-1* were detected in a clinical sample of oesophageal carcinoma (Case E-1). Oesophageal carcinoma expressed 2.1 CTA genes on average, the largest number among the 4 types of carcinomas examined in this study. In oesophageal carcinoma, 39 (84.7%) of 46 samples expressed at least 1 CTA gene and 28 (60.9%) of 46 samples expressed at least 2 CTA genes synchronously. In colorectal carcinoma, 25 (20.4%) of 98 samples expressed at least 1 CTA gene, and only 5 (5.1%) of 98 samples expressed multiple CTA genes synchronously. The results for gastric and breast carcinoma are summarized in Figure 2B.

### Synchronous expression of CTA genes with *MAGE* genes

We analyzed the synchronous expression of CTA genes with *MAGE-1* or *MAGE-3* gene. A total of 18 (46.2%) of 39 oesophageal carcinoma samples expressed *LAGE-1* with synchronous expression of *MAGE-1* or *MAGE-3* genes, 21 (46.7%) of 45 gastric carcinomas synchronously expressed *SSX-4*, and 18

**Table 2** Expression of CTA genes in gastrointestinal and breast carcinomas

| | NY-ESO-1 (%) | LAGE-1 (%) | SCP-1 (%) | SSX-1 (%) | SSX-2 (%) | SSX-4 |
|---|---|---|---|---|---|---|
| Oesophageal carcinoma<br>*n* = 46 | 11<br>(23.9) | 18<br>(39.1) | 1<br>(2.2) | 0<br>(0) | 0<br>(0) | 6<br>(13.0) |
| Gastric carcinoma<br>*n* = 102 | 8<br>(7.8) | 12<br>(11.8) | 24<br>(23.5) | 3<br>(2.9) | 3<br>(2.9) | 21<br>(20.6) |
| Colorectal carcinoma<br>*n* = 98 | 2<br>(2.0) | 3<br>(3.1) | 0<br>(0) | 0<br>(0) | 2<br>(2.0) | 2<br>(2.0) |
| Breast carcinoma<br>*n* = 129 | 13<br>(10.1) | 4<br>(3.1) | 44<br>(34.1) | 1<br>(0.8) | 5<br>(3.9) | 12<br>(9.3) |

 *© 2001 Cancer Research Campaign*

BJOC 01-1974 713-720 20/8/01 4:20 pm Page 717

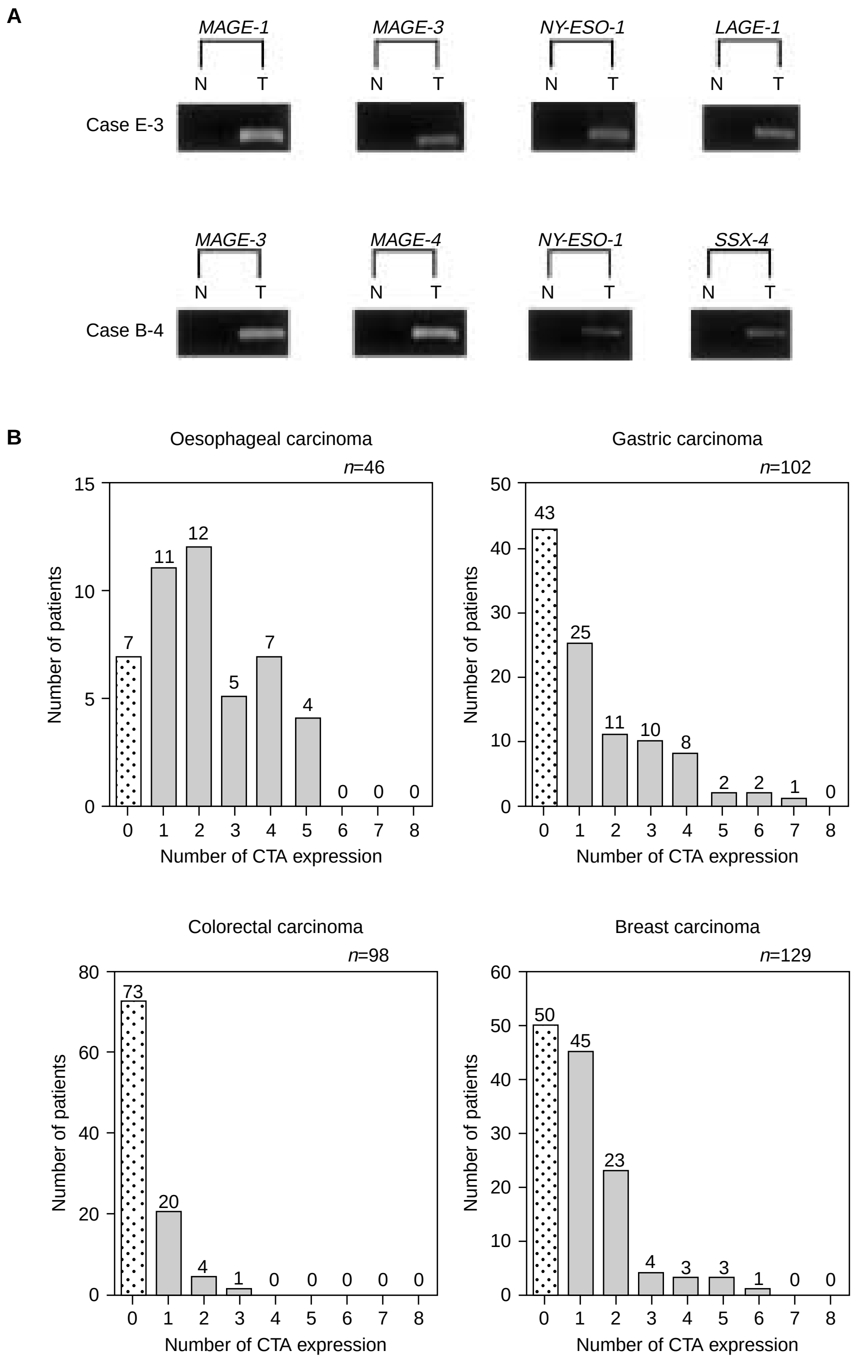

**Figure 2** (**A**) Synchronous expression of multiple CTA genes in the oesophageal and breast carcinoma cases detected by RT-PCR. Case E-3, oesophageal carcinoma with expression of *MAGE-1*, *MAGE-3*, *NY-ESO-1*, and *LAGE-1*. Case B-4, breast carcinoma with expression of *MAGE-3*, *MAGE-4*, *NY-ESO-1*, and *SSX-4*. cDNA extracted from tumour tissue (T) and paired normal tissue (N) were amplified in each case. (**B**) Expression of 8 CTA genes (*MAGE-1*, *MAGE-3*, *NY-ESO-1*, *LAGE-1*, *SCP-1*, *SSX-1*, *SSX-2*, *SSX-4*) in 46 oesophageal carcinomas, 102 gastric carcinomas, 98 colorectal carcinomas, and 129 breast carcinomas. Number of CTA genes expressed per clinical sample is summarized. Synchronous expression of multiple CTA genes was detected in 28 (61%) of 46 oesophageal carcinoma samples, 34 (33%) of 102 gastric carcinoma samples and 34 (26%) of 129 breast carcinoma samples

(38.3%) of 47 breast carcinomas synchronously expressed *SCP-1*. We also analysed the cases with expression of neither *MAGE-1* nor *MAGE-3* genes to determine targets for cancer immunotherapy other than *MAGE* genes. A total of 26 (31.7%) of 82 samples of breast carcinoma and 12 (21.1%) of 57 samples of gastric carcinoma expressed the *SCP-1* gene, with expression of neither *MAGE-1* nor *MAGE-3*. These results are summarized in Figure 3.

### Differential distribution of carcinoma cells staining with CTA protein-specific mAbs in carcinoma tissues

We investigated the distribution of carcinoma cells expressing CTA proteins in tumour tissues by immunohistochemical analysis for representative cases. The primary antibodies used in this study were 57B, a murine mAb against MAGE-4 protein (Landry et al,

© 2001 Cancer Research Campaign

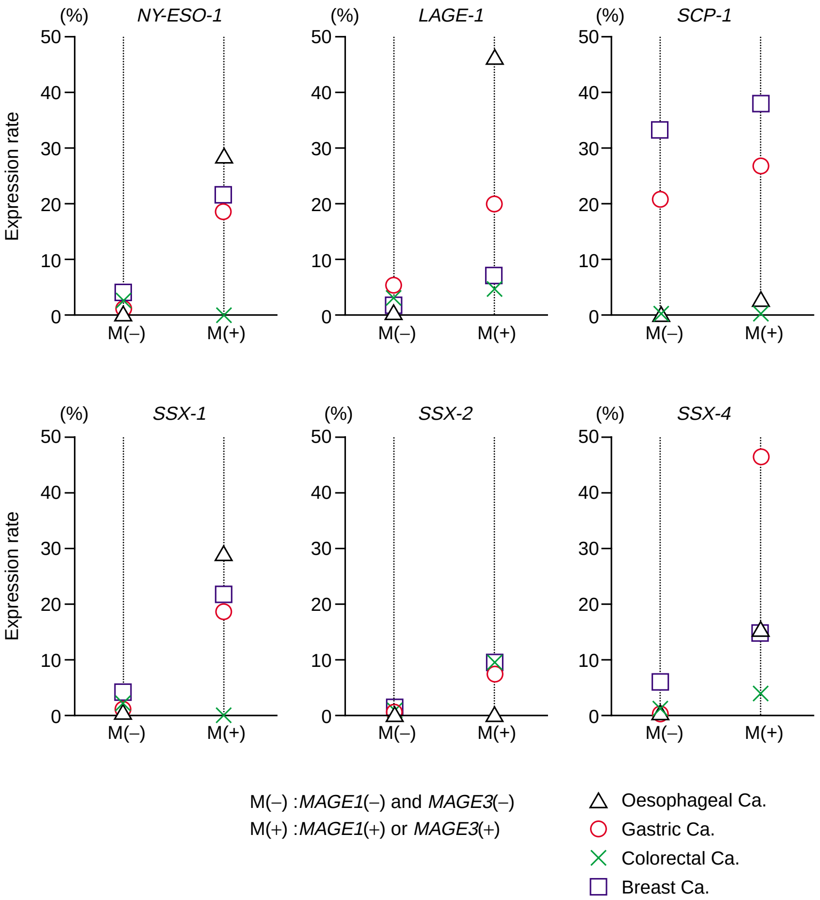

**Figure 3** Comparison of CTA gene expression between *MAGE-1*-or *MAGE-3*-positive cases, M (+) and both the *MAGE-1*- and *MAGE-3*-negative cases, M (–). *MAGE*-positive cases tended to express the other CTAs frequently. *LAGE-1* in oesophageal carcinoma, *SSX-4* in gastric carcinoma and *SCP-1* in breast carcinoma were synchronously expressed with *MAGE* genes frequently. *SCP-1* tended to be expressed in both *MAGE*-positive and -negative cases

2000), and B9.8, a murine mAb against NY-ESO-1 protein (Schultz et al, 2000). Case B-4 was one of breast carcinoma, in which *MAGE-3*, *MAGE-4*, *NY-ESO-1*, and *SSX-4* were detected in RT-PCR analysis (Figure 2A). Heterogeneity of 57B and B9.8 staining was detected in the tumour specimens of Case B-4. The staining, which was mostly detectable in the cell cytoplasm, appeared to be limited to carcinoma cells. The tumour cells stained with 57B and B9.8 revealed the differential distribution of MAGE-4 and NY-ESO-1 in breast carcinoma tissues (Figure 4). We examined 5 cases expressing both *MAGE-4* and *NY-ESO-1* genes and revealed the differential distribution in 2 cases and the same distribution in 3 cases.

### DISCUSSION

In our institute, clinical trials of cancer-specific immunotherapy have been ongoing on patients with gastrointestinal carcinomas, using antigen-presenting cells pulsed with MAGE peptides. Good results were observed in some clinical cases, but there are two problems that need to be solved. One of the problems is the restriction of candidates for immunotherapy. The prerequisite for therapy is *MAGE* gene expressions in tumour tissues and adaptation of patient HLA type to peptide-binding specificity. Another is the heterogeneous expression pattern of MAGE, which may be a strategy for evasion of immunosurveillance by malignant cells (Jungbluth et al, 2000; Sadanaga et al, 1999). In the present study, we found 3 novel possibilities for a solution of these problems.

First, we found high expression of CTAs other than *MAGE-1* or *MAGE-3* in gastric and oesophageal carcinomas. We found high expression of *SCP-1* (23%) and *SSX-4* (20%) genes in gastric carcinoma and of *NY-ESO-1* (19%) and *LAGE-1* (38%) genes in oesophageal carcinoma. High expression of *SSX* genes was detected in a wide variety of neoplasms such as head and neck carcinomas, colorectal carcinoma, and breast carcinoma (Türeci et al, 1998a). We also found high expression of *SSX-4* in the gastric carcinoma, in which either the *MAGE-1* or *MAGE-3* gene was co-expressed. These findings suggest that these antigens might be targets for cancer-specific immunotherapy in addition to MAGE-1 and MAGE-3. *NY-ESO-1* and *LAGE-1* were also reported to be expressed in a large number of breast carcinomas (Chen et al, 1997; Lethe et al, 1998), but we could not detect a high rate of expression of either gene in breast carcinomas. These differences might potentially contribute to the disparity in survival rate after breast cancer among the various races (Dignam, 2000).

Secondly, we found high expression of *SCP-1* in cases expressing neither MAGE-1 nor MAGE-3 in gastric and breast carcinomas. The *SCP-1* gene was expressed in 26 (31.7%) of the 82 samples of breast carcinoma expressing neither *MAGE-1* nor *MAGE-3*, and was expressed in 12 (21.2%) of 57 samples of gastric carcinoma expressing neither *MAGE-1* nor *MAGE-3*. In

 *© 2001 Cancer Research Campaign*

BJOC 01-1974 713-720 20/8/01 4:20 pm Page 719

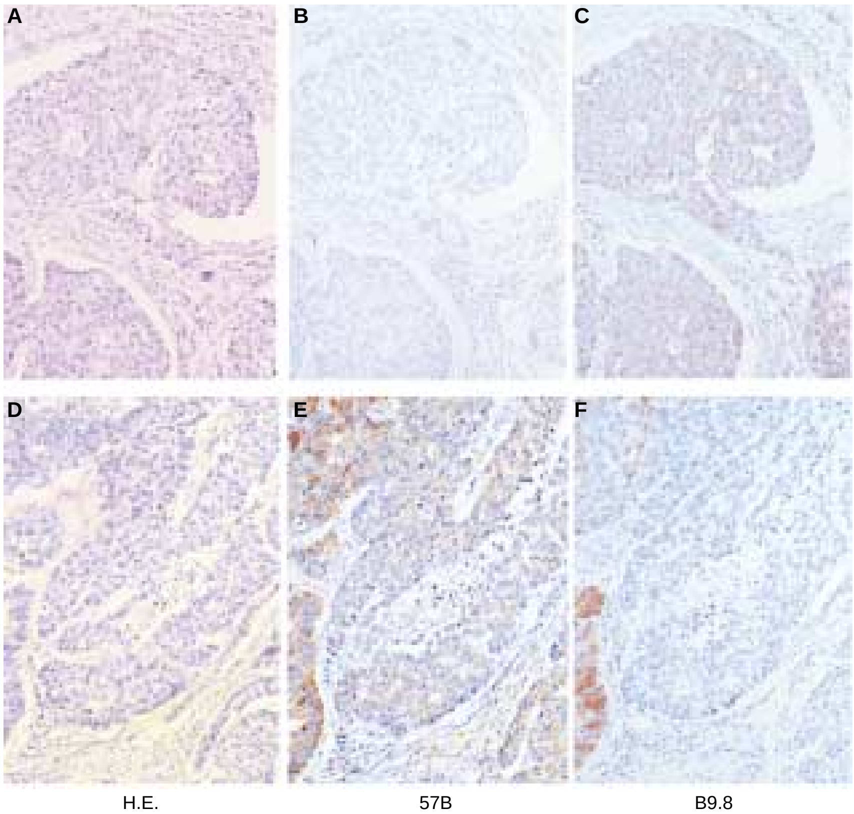

**Figure 4** Immunohistochemical and RT-PCR analysis of cases expressing multiple CTAs. As shown in Figure 2A, RT-PCR analysis revealed synchronous expression of *MAGE-3*, *MAGE-4*, *NY-ESO-1*, and *SSX-4* in this breast carcinoma case, Case B-4. Immunohistochemical staining with 57B and B9.8, consecutive sections of Case B-4 (**A–F**). The upper panels show NY-ESO-1 protein immunoreactivity of B9.8 mAb (**C**) was detected in the carcinoma cells not expressing the MAGE-4 gene product (**B**). The lower panels show MAGE-4 protein immunoreactivity of 57B (**E**) was detected in the carcinoma cells not expressing NY-ESO-1 gene product (**F**). Haematoxylin-eosin staining of the sections is shown in (**A**) and (**D**). (Original magnification × 200)

addition, *SCP-1* was expressed more frequently than *MAGE-1* and *MAGE-3* in breast carcinoma. These results suggest that if *SCP-1* is a new target antigen in addition to *MAGE-1* and *MAGE-3*, the candidates for CTA-based cancer immunotherapy for gastric and breast carcinomas will be increased in number. *SCP-1* was identified as HOM-TES-14, during screening of a cDNA library enriched for testis-specific clones with serum from a renal cell carcinoma patient (Türeci et al, 1998b). High levels of expression of *SCP-1* in tumour tissue and normal testis are recognized at the mRNA and protein levels by RT-PCR and Western blot analysis, respectively. The antigenic peptides encoded by these CTAs will be identified in future studies. Patients' opportunities for cancer-specific immunotherapy can thus be expanded.

Thirdly, we found a high frequency of synchronous expression of multiple CTA genes in clinical tumour samples. At least 2 CTA genes were expressed in 28 (61%) of 46 oesophageal carcinomas, 34 (33%) of 102 gastric carcinomas, and 34 (26%) of 129 breast carcinomas (Figure 3). In the present study, we examined representative samples of breast carcinoma with immunohistochemical analysis of 57B or B9.8 antibodies. Antibody 57B stains the tumours that express the *MAGE-4* gene, and antibody B9.8 recognized tumours that expressed the *NY-ESO-1* gene (Landry et al, 2000; Schultz et al, 2000). As shown in Figure 2A and Figure 4, Case B-4 expressed both *MAGE-4* and *NY-ESO-1*, and immunohistochemical analysis revealed differential distribution of MAGE-4-or NY-ESO-1-positive carcinoma cells. We and others have reported heterogeneous expression of *MAGE* genes in tumour tissues using immunohistochemical analysis (Jungbluth et al, 2000; Sadanaga et al, 1999). In this study, we found the heterogeneous expression of MAGE-4 and NY-ESO-1 in carcinoma tissues, indicating that some parts of carcinoma cells were stained with 57B antibody, and others with B9.8 antibody (Figure 4). This differential distribution of staining of carcinoma cells indicates that the area of carcinoma cells expressing tumour rejection antigens might be enlarged. Therefore, in this case, a combined vaccination based on both MAGE-4 and NY-ESO-1 may cause immunological reactions in a larger area of tumour tissues than a single peptide-based vaccination. Taken together, our findings suggest that polyvalent vaccinations with multiple antigens might be necessary to obtain good clinical results in cancer immunotherapy.

Despite shared expression of multiple CTAs in tumour species, individual antigens exhibit distinct variation in quantity. Immunohistochemical analysis revealed heterogeneous expression, and RT-PCR analysis revealed distinct intensity of a band in agarose gels. It is important to evaluate the quantity of expression of each antigen when use of immunotherapy is decided.

In conclusion, the predominant expression of *NY-ESO-1* and *LAGE-1* in oesophageal carcinoma, *SSX-4* and *SCP-1* in gastric carcinoma and *SCP-1* in breast carcinoma suggest possible targets for cancer-specific immunotherapy in addition to *MAGE-1* and *MAGE-3*. Furthermore, *SCP-1* could be a novel target for patients

*© 2001 Cancer Research Campaign* 

without expression of *MAGE-1* and *MAGE-3* genes in gastric and breast carcinomas. We also demonstrate the possibility that polyvalent vaccination with multiple CTA genes will yield effective clinical results for cancer-specific immunotherapy.

### ACKNOWLEDGEMENTS

We thank Dr Bernard Lethe for kindly providing the melanoma cell line LB373-MEL, and Dr G C Spagnoli for kindly providing the CTA-specific antibodies. We are also grateful to Mr K Satoh, Ms J Miyake, Ms K Ogata, and Ms T Shimooka for their excellent technical assistance.

### REFERENCES

Boel P, Wildmann C, Sensi ML, Brasseur R, Renauld JC, Coulie P, Boon T and van der Bruggen P (1995) BAGE: a new gene encoding an antigen recognized on human melanomas by cytolytic T lymphocytes. *Immunity* **2**: 167–175

Chaux P, Vantomme V, Stroobant V, Thielemans K, Corthals J, Luiten R, Eggermont AM, Boon T and van der Bruggen P (1999) Identification of MAGE-3 epitopes presented by HLA-DR molecules to CD4(+) T lymphocytes. *J Exp Med* **189**: 767–778

Chen YT, Gure AO, Tsang S, Stockert E, Jager E, Knuth A and Old LJ (1998) Identification of multiple cancer/testis antigens by allogeneic antibody screening of a melanoma cell line library. *Proc Natl Acad Sci USA* **95**: 6919–6923

Chen YT, Scanlan MJ, Sahin U, Türeci O, Gure AO, Tsang S, Williamson B, Stockert E, Pfreundschuh M and Old LJ (1997) A testicular antigen aberrantly expressed in human cancers detected by autologous antibody screening. *Proc Natl Acad Sci USA* **94**: 1914–1918

Chomczynski P and Sacchi N (1987) Single-step method of RNA isolation by acid guanidinium thiocyanate-phenol-chloroform extraction. *Anal Biochem* **162**: 156–159

Dignam JJ (2000) Differences in breast cancer prognosis among African-American and Caucasian women. *CA Cancer J Clin* **50**: 50–64

Fujie T, Mori M, Ueo H, Sugimachi K and Akiyoshi T (1997) Expression of MAGE and BAGE genes in Japanese breast cancers. *Ann Oncol* **8**: 369–372

Fujie T, Tahara K, Tanaka F, Mori M, Takesako K and Akiyoshi T (1999) A MAGE-1-encoded HLA-A24-binding synthetic peptide induces specific anti-tumor cytotoxic T lymphocytes. *Int J Cancer* **80**: 169–172

Greenlee RT, Murray T, Bolden S and Wingo PA (2000) Cancer statistics, 2000. *CA Cancer J Clin* **50**: 7–33

Gure AO, Türeci O, Sahin U, Tsang S, Scanlan MJ, Jager E, Knuth A, Pfreundschuh M, Old LJ and Chen YT (1997) SSX: a multigene family with several members transcribed in normal testis and human cancer. *Int J Cancer* **72**: 965–971

Inoue H, Mori M, Honda M, Li J, Shibuta K, Mimori K, Ueo H and Akiyoshi T (1995) The expression of tumor-rejection antigen ‘MAGE’ genes in human gastric carcinoma. *Gastroenterology* **109**: 1522–1525

Inoue K, Ozeki Y, Suganuma T, Sugiura, Y and Tanaka S (1997) Vascular endothelial growth factor expression in primary esophageal squamous cell carcinoma. Association with angiogenesis and tumor progression. *Cancer* **79**: 206–213

Jungbluth AA, Stockert E, Chen YT, Kolb D, Iversen K, Coplan K, Williamson B, Altorki N, Busam KJ and Old LJ (2000) Monoclonal antibody MA454 reveals a heterogeneous expression pattern of MAGE-1 antigen in formalin-fixed paraffin embedded lung tumours. *Br J Cancer* **83**: 493–497

Kocher T, Schultz TE, Gudat F, Schaefer C, Casorati G, Juretic A, Willimann T, Harder F, Heberer M and Spagnoli GC (1995) Identification and intracellular location of MAGE-3 gene product. *Cancer Res* **55**: 2236–2239

Landry C, Brasseur F, Spagnoli GC, Marbaix E, Boon T, Coulie P and Godelaine D (2000) Monoclonal antibody 57B stains tumor tissues that express gene MAGE-A4. *Int J Cancer* **86**: 835–841

Lethe B, Lucas S, Michaux L, De SC, Godelaine D, Serrano A, De PE and Boon T (1998) LAGE-1, a new gene with tumor specificity. *Int J Cancer* **76**: 903–908

Manici S, Sturniolo T, Imro MA, Hammer J, Sinigaglia F, Noppen C, Spagnoli G, Mazzi B, Bellone M, Dellabona P and Protti MP (1999) Melanoma cells present a MAGE-3 epitope to CD4(+) cytotoxic T cells in association with histocompatibility leukocyte antigen DR11. *J Exp Med* **189**: 871–876

Marchand M, Weynants P, Rankin E, Arienti F, Belli F, Parmiani G, Cascinelli N, Bourlond A, Vanwijck R, Humblet Y, Canon J-L, Laurent C, Naeyaert J-M, Plagne R, Deraemaeker R, Knuth A, Jager E, Brasseur F, Herman J, Coulie PG and Boon T (1995) Tumor regression responses in melanoma patients treated with a peptide encoded by gene MAGE-3. *Int J Cancer* **63**: 883–885

Mori M, Inoue H, Mimori K, Shibuta K, Baba K, Nakashima H, Haraguchi M, Tsuji K, Ueo H, Barnard GF and Akiyoshi T (1996) Expression of MAGE genes in human colorectal carcinoma. *Ann Surg* **224**: 183–188

Mukherji B, Chakraborty NG, Yamasaki S, Okino T, Yamase H, Sporn JR, Kurtzman SK, Ergin MT, Ozols J, Meehan J and Murai F (1995) Induction of antigen-specific cytolytic T cells in situ in human melanoma by immunization with synthetic peptide-pulsed autologous antigen presenting cells. *Proc Natl Acad Sci USA* **92**: 8078–8082

Ries LA, Wingo PA, Miller DS, Howe HL, Weir HK, Rosenberg HM, Vernon SW, Cronin K and Edwards BK (2000) The annual report to the nation on the status of cancer, 1973–1997, with a special section on colorectal cancer. *Cancer* **88**: 2398–2424

Sadanaga N, Nagashima H, Tahara K, Yoshikawa Y and Mori M (1999) The heterogeneous expression of MAGE-3 protein: difference between primary lesions and metastatic lymph nodes in gastric carcinoma. *Oncol Rep* **6**: 975–977

Sahin U, Türeci O, Schmitt H, Cochlovius B, Johannes T, Schmits R, Stenner F, Luo G, Schobert I and Pfreundschuh M (1995) Human neoplasms elicit multiple specific immune responses in the autologous host. *Proc Natl Acad Sci USA* **92**: 11810–11813

Schultz TE, Noppen C, Gudat F, Durmuller U, Zajac P, Kocher T, Heberer M and Spagnoli GC (2000) NY-ESO-1 tumour associated antigen is a cytoplasmic protein detectable by specific monoclonal antibodies in cell lines and clinical specimens. *Br J Cancer* **83**: 204–208

Tahara K, Mori M, Sadanaga N, Sakamoto Y, Kitano S and Makuuchi M (1999) Expression of the MAGE gene family in human hepatocellular carcinoma. *Cancer* **85**: 1234–1240

Tanaka F, Fujie T, Tahara K, Mori M, Takesako K, Sette A, Celis E and Akiyoshi T (1997) Induction of antitumor cytotoxic T lymphocytes with a MAGE-3-encoded synthetic peptide presented by human leukocytes antigen-A24. *Cancer Res* **57**: 4465–4468

Tokunaga K, Nakamura Y, Sakata K, Fujimori K, Ohkubo M, Sawada K and Sakiyama S (1987) Enhanced expression of a glyceraldehyde-3-phosphate dehydrogenase gene in human lung cancers. *Cancer Res* **47**: 5616–5619

Türeci O, Chen YT, Sahin U, Gure AO, Zwick C, Villena C, Tsang S, Seitz G, Old LJ and Pfreundschuh M (1998a) Expression of SSX genes in human tumors. *Int J Cancer* **77**: 19–23

Türeci O, Sahin U, Schobert I, Koslowski M, Scmitt H, Schild HJ, Stenner F, Seitz G, Rammensee HG and Pfreundschuh M (1996) The SSX-2 gene, which is involved in the t(X;18) translocation of synovial sarcomas, codes for the human tumor antigen HOM-MEL-40. *Cancer Res* **56**: 4766–4772

Türeci O, Sahin U, Zwick C, Koslowski M, Seitz G and Pfreundschuh M (1998b) Identification of a meiosis-specific protein as a member of the class of cancer/testis antigens. *Proc Natl Acad Sci USA* **95**: 5211–5216

Van den Eynde B, Peeters O, De BO, Gaugler B, Lucas S and Boon T (1995) A new family of genes coding for an antigen recognized by autologous cytolytic T lymphocytes on a human melanoma. *J Exp Med* **182**: 689–698

Van den Eynde B and Boon T (1997) Tumor antigens recognized by T lymphocytes. *Int J Clin Lab Res* **27**: 81–86

Van der Bruggen P, Traversari C, Chomez P, Lurquin C, De PE, Van den Eynde B, Knuth A and Boon T (1991) A gene encoding an antigen recognized by cytolytic T lymphocytes on a human melanoma. *Science* **254**: 1643–1647

Weynants P, Lethe B, Brasseur F, Marchand M and Boon T (1994) Expression of mage genes by non-small-cell lung carcinomas. *Int J Cancer* **56**: 826–829

 *© 2001 Cancer Research Campaign*
